# Augmented und Virtual Reality in der Chirurgie: Einsatzgebiete und explorative Studien am Beispiel von VIVATOP: Perioperative Operationsplanung und intraoperative Unterstützung

**DOI:** 10.1007/s00104-024-02218-3

**Published:** 2025-01-03

**Authors:** Dirk Weyhe, Verena Hartmann, Verena Uslar, Navid Tabriz

**Affiliations:** Pius-Hospital Oldenburg, Universitätsklinik für Viszeralchirurgie, Universitätsmedizin Oldenburg, Georgstr. 12, 26121 Oldenburg, Deutschland

**Keywords:** Digitale Technologien, Viszeralchirurgie, 3‑D-Druck, Telemedizin, Avatar, Digital technologies, Visceral surgery, 3D printing, Telemedicine, Avatar

## Abstract

Digitale Technologien wie Virtual und Augmented Reality (VR/AR) werden in der präklinischen und klinischen Phase vor allem in der Neurochirurgie sowie in der Orthopädie angewendet. Dagegen ist in der Viszeralchirurgie die Anwendung seltener, da die intraoperativen Deformationen herausfordernd für den klinischen Einsatz sind. Vor allem in der Aus- und Weiterbildung werden VR-Anwendungen erfolgreich eingesetzt. Neben aktuellen Anwendungsgebieten beleuchtet dieser Beitrag Ergebnisse des Projekts VIVATOP (Vielseitiger Immersiver Virtueller und Augmentierter Tangible OP) des Bundesministeriums für Bildung und Forschung (BMBF). Hier wurden AR- und VR-Technologien in Kombination mit 3‑D-Druck als Demonstratoren neu- bzw. weiterentwickelt. Es erfolgte die Entwicklung eines VR-Planungswerkzeugs zur Leberteilresektion, die Entwicklung von 3‑D-Hologrammen zur intraoperativen AR-Unterstützung und eine Avatartelemedizinfunktion sowie ein 3‑D-Druck-Trainingsmodell. Die klinischen Ergebnisse der intraoperativen AR-Unterstützung mit dem primären Endpunkt Operationsdauer und den sekundären Endpunkten Krankenhaus- und Intensivaufenthalt sowie Komplikationsraten werden mit einer historischen Kohorte verglichen und die Ergebnisse kontextualisiert.

## Abstract (en)

Digital technologies, such as virtual and augmented reality (VR and AR) are mainly used in the preclinical and clinical phases in neurosurgery and orthopedics. In contrast, they are used less frequently in visceral surgery as the intraoperative deformation is challenging for the clinical use. The application of VR is used successfully particularly in education and training. In addition to current areas of application, this article highlights the results of the Federal Ministry of Education and Research (BMBF) project “Versatile Immersive Virtual and Augmented Tangible OP (= surgery)” (VIVATOP). In this project AR and VR technologies in combination with 3D printing as demonstrators were newly or further developed. A VR planning tool for partial liver resection, the development of 3D holograms for intraoperative AR support and an avatar telemedicine function as well as a 3D printed model for training purposes were developed. The clinical results of the intraoperative AR support with the primary endpoint of operation duration and the secondary endpoints of the duration of hospitalization and intensive care unit stay as well as complication rates are compared with a historical cohort and the results are contextualized.

Augmented und Virtual Reality sind Zukunftstechnologien mit großem Potenzial im klinischen Umfeld. Bisher konnte ein Mehrwert der Technologien bereits in der Aus- und Weiterbildung nachgewiesen werden. Ein evidenzbasierter Mehrwert im klinischen Umfeld konnte bisher nicht gezeigt werden. Die vorliegende Arbeit führt in die klinische Thematik ein und stellt die klinischen Evaluationsergebnisse der in „VIVATOP“ entwickelten Demonstratoren vor.

## Virtual und Augmented Reality: aktuelle chirurgische Einsatzgebiete

Digitale Technologien wie Virtual Reality (VR) und Augmented Reality (AR) kommen in der präklinischen und klinischen Phase bisher vor allem in der Neurochirurgie sowie in der Orthopädie zum Einsatz. Die prä- und intraoperative Bildgebung in der Orthopädie mit relativ starren Strukturen wie Muskelverläufe und Knochen führen dazu, dass die AR-Technologien in der Orthopädie als sog. „Beachhead“-Markt in der operativen Medizin eingeschätzt wird [1]. Dagegen ist in der Viszeralchirurgie die verfügbare Literatur mit 4 % Publikationsanteil eher gering, da die intraoperativen Deformationen anders als in der Neurochirurgie und Orthopädie besonders herausfordernd für den klinischen Einsatz sind [2]. Vor allem im präklinischen Umfeld werden zur Aus- und Weiterbildung diese digitalen Unterstützungssysteme zumeist als VR-Anwendungen schon vielfältig eingesetzt [3–6]. Klinische Einsatzbereiche sind dagegen selten und meist nur als kleine Fallserien beschrieben [7, 8].

Im vorliegenden Artikel werden zunächst ausführlich aktuelle Anwendungsgebiete in operativen Fächern sowie das durch das Bundesministerium für Bildung und Forschung (BMBF) geförderte Projekt Vielseitiger Immersiver Virtueller und Augmentierter Tangible OP (VIVATOP) dargestellt, in dem AR- und VR-Technologien in Kombination mit 3‑D-Druck für die Viszeralchirurgie neu- oder weiterentwickelt wurden [9]. Des Weiteren werden die Ergebnisse der klinischen Erprobung der entwickelten Demonstratoren im Rahmen von Leberresektionen an *n* = 11 Patientinnen und Patienten beschrieben und mit einem historischen Patientenkollektiv verglichen.

### Neurochirurgie

Van Gestel et al. fanden bei der Platzierung externer ventrikulärer Drainagen unter AR-Navigation im Vergleich zur Freihandtechnik eine höhere Präzisionsgenauigkeit und auch eine höhere Ergebnisqualität selbst für Verfahrensanfänger [10]. Die AR-gestützte Platzierung wurde in ihrer Studie von unerfahrenen und erfahrenen Probanden an Phantommodellen durchgeführt. Dabei verbesserte die AR-Anwendung die Ergebnisleistung positiv und führte zu einer Verbesserung der Lernkurve. Diese AR-gestützten Verfahren sollen nun in das klinische Umfeld übertragen werden. Außerdem werden AR-basierte Planungen zur Resektion intrakranieller Tumoren von der gleichen Arbeitsgruppe bereits erfolgreich eingesetzt [11]. Mit der Entwicklung eines AR-Navigationssystems, das in Kombination mit der HoloLens II (Microsoft Corporation, Redmond, WA, USA) eingesetzt wurde, entsprach die Registrierungsgenauigkeit einem hochmodernen Neuronavigationssystem. Durch die Bereitstellung einer intuitiveren Visualisierung relevanter Daten für den Neurochirurgen hat der entwickelte AR-Navigationsworkflow zu einer genauen Methode für die Tumorresektionsplanung, mit detaillierteren Tumorabgrenzungen und einer reduzierten präoperativen Planungszeit in der genannten Studiengruppe geführt.

### Orthopädie

In einem Review konnte gezeigt werden, dass viele AR-Technologien einerseits die kognitive Belastung des Anwenders senken und zugleich die Operationszeit sowie die Strahlenexposition reduzieren [1]. Gleichzeitig wird die chirurgische Präzision in präklinischen Kadaver- und Sägeknochenmodellen verbessert. Bisher wurden nur wenige klinisch anwendbare Plattformen, die sich z. B. auf die Platzierung von Pedikelschrauben konzentrieren, von der United States Food and Drug Administration (FDA) genehmigt.

Eine randomisierte verblindete Studie untersuchte die Effektivität von VR-Training in der Orthopädie [12]. Die Ergebnisse zeigten, dass VR-gestütztes Training signifikant bessere Ergebnisse in Bezug auf Präzision und Geschwindigkeit der chirurgischen Eingriffe ermöglichen kann. Aber auch in der Orthopädie, bei der nicht die Problematik der Organdeformation besteht, bleibt laut Ghaednia et al. abzuwarten, ob die aktuellen digitalen Technologien die Vielzahl der Erwartungen erfüllen können [13].

### Viszeralchirurgie

Trotz der Deformationsproblematik haben auch in der Viszeralchirurgie AR, VR bzw. Mixed Reality (MR) ein großes perioperatives Potenzial und werden als digitale Assistenzsysteme zukünftig ihren Stellewert im klinischen Alltag finden. Lang et al. berichteten erstmals 2005 über die Auswirkungen der virtuellen Tumorresektion und der computergestützten Risikoanalyse auf die Operationsplanung und die intraoperative Strategie bei größeren Leberresektionen [14]. Während diese Planungen zunächst zweidimensional am PC-Bildschirm erfolgten, wurden im Verlauf der Jahre sog. Head Mounted Displays (HMDs) wie z. B. das HoloLens HMD als Endgerät mit den Möglichkeiten einer interaktiven dreidimensionalen Planung eingeführt [15]. Quero et al. setzten dann 2018 die Technologie für Leberresektionen ein [8], Lau et al. 2019 als präklinische Evaluation der weiterentwickelten Technologie [16].

Digitale Assistenzsysteme haben das Potenzial, chirurgische Strategien umzugestalten

Neuere Publikationen zeigen in der klinischen AR-Anwendung intraoperative Vorteile [17]. Bei einer Fallserie von 8 Leberoperationen wurden zur Rekonstruktion ein Open-source-Softwarepaket zur Darstellung die VSI-Holomedicine-Software auf der Microsoft-Hololens genutzt. Die Autoren beschreiben zur Vorbereitungszeit für die Erstellung der Hologramme einen zeitlichen Mehraufwand von 150–360 min. Intraoperativ bestand aber für das gesamte Operationsteam der subjektive Eindruck, mehr Information zur Lagebeziehung anatomischer Strukturen zueinander wie z. B. portalvenöses System und der Lage der Tumoren zu erhalten. Weitere erfolgreiche Einsätze sind auch bei der komplexen Leberchirurgie wie z. B. der ALPPS („associating liver partition and portal vein ligation for staged hepatectomy“; assoziierte Leberpartition und Pfortaderligatur bei gestufter Hepatektomie) allerdings nur kasuistisch beschrieben [18]. Die digitalen Assistenzsysteme haben das Potenzial einen Paradigmenwechsel einzuleiten, der chirurgische Strategien, Trainingsmethoden und Patientenaufklärung umgestalten kann [19].

Vor diesem Hintergrund wurde in dem BMBF-geförderten Projekt VIVATOP die wechselseitige Beziehung zwischen neu entwickelten immersiven Technologiedemonstratoren, dem 3‑D-Druck und Anwendungsmöglichkeiten am Beispiel parenchymerhaltender Leberoperationen evaluiert.

## VIVATOP

Obwohl die Segmentierung von DICOM(Digital Imaging and Communications in Medicine)-Daten am Beispiel der Leber bereits seit mehr als 20 Jahren technisch möglich ist, hat diese Technik im klinischen Alltag bisher keine höhere Relevanz eingenommen.

Vor diesem Hintergrund wurde das BMBF-geförderte Projekt Vielseitiger Immersiver und Augmentierter Tangible OP (VIVATOP) unter der Leitung des Technologiezentrums für Informatik und Informationstechnik (TZI) Bremen (Prof. Malaka) beantragt, um AR und VR als immersive Technologien mit patientenrealistischen 3‑D-Druckmodellen weiterzuentwickeln, um dann die Demonstratoren auf ihr Anwendungspotenzial klinisch zu evaluieren [9]. Die Weiterentwicklung der Segmentierungssoftware zur Planung in VR und intraoperativen Unterstützung im Sterilfeld in AR sowie die Zusammenführung immersiver Technologien und 3‑D-Druck zum Schließen der haptischen Lücke und die Evaluation der entstandenen Demonstratoren waren die übergeordneten Ziele des Projektes (www.vivatop.de).

### Präoperative Planung

**Abb. 1:**
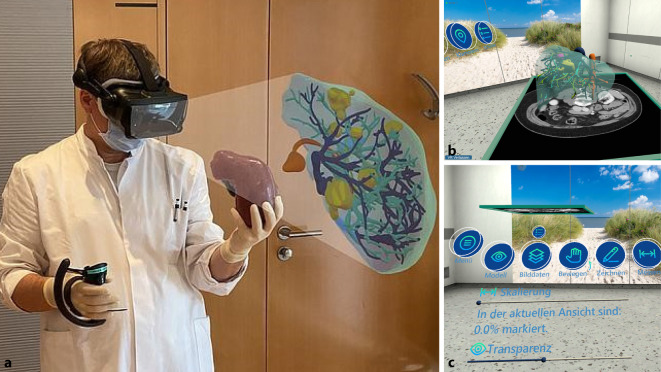
**a** Nutzer des Operationsplanungstools mit haptisch korrektem Lebercontroller (linke Hand des Nutzers); die Position der in Virtual Reality (VR) betrachteten Leber orientiert sich an der Position des Controllers (s. eingeblendete Ansicht aus der VR-Umgebung); **b**, **c** Screenshots der VR-Umgebung

Zur präoperativen Planung der Leberresektion wurde im Projekt ein VR-Planungstool entwickelt. Dieses Planungstool kam zur verbesserten Immersion in Kombination mit einer 3‑D-Druck-Leber als haptischer Kontroller zur Anwendung (Abb. 1a). In der Arbeit von Münder et al. wurden die Auswirkungen von weichem und festem Material in einer Nutzerstudie mit 14 Chirurginnen und Chirurgen untersucht. Verglichen wurden zwei Materialien (weich/fest) von 3‑D-Druck-Lebern [20]. Die Ergebnisse in der Planungsinteraktion zeigen eine klare Präferenz für das weichere Material, da es als realistischer empfunden wurde. Zudem zeigte sich, dass der haptische Einfluss des 3‑D-Drucks mit 75 % der Organgröße und weiche 3‑D-Druck-Modelle zu einer deutlich stärkeren Immersion im Rahmen der Operationsplanung führte [21]. In die neuentwickelte Software der VR-Umgebung wurden die vom Fraunhofer-Institut MEVIS segmentierten Lebermodelle eingebettet (Abb. 1b, c). In diesem Setting erfolgte dann die präoperative Planung der Leberresektion [22]. Die Teilnehmer benötigten durchschnittlich 14,3 min (SD = 3,59), um die Fälle in VR-Umgebung zu planen.

Reinschüssel et al. führten vor und nach der Operation halbstrukturierte Interviews zur Evaluation von Vor- und Nachteilen der VR-Operationsplanung durch. Die Benutzerfreundlichkeit war dabei als gut beschrieben worden. Die Ergebnisse aus den Interviews und Beobachtungen sehen deutliche Vorteile in der VR-Planung und das Potenzial, die Patientensicherheit zu verbessern. Das eine hohe zeitliche Effizienz der Planung mit digitalen Assistenzsystemen möglich ist, zeigt eine vergleichende Studie im Cross-over-Design, in der die AR-Planung mit der konventionellen Planung in der 2‑D-Computertomographie(CT)/Magnetresonanztomographie(MRT)-Standarddiagnostik erfolgte [23].

### Intraoperative Unterstützung

**Abb. 2:**
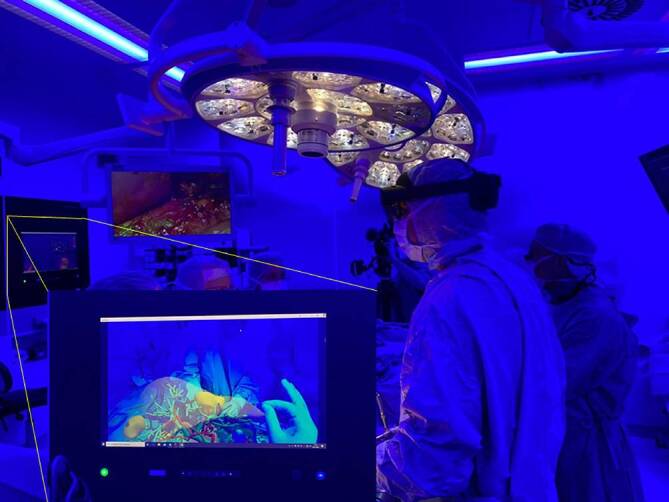
Anwendung des im VIVATOP-Projekt entwickelten AR(Augmented Reylity)-Demonstrators im Operationssaal. Zur besseren Kontrastierung des 3‑D-Modells wird blaues Umgebungslicht genutzt

Zur intraoperativen Entscheidungsunterstützung wurden dem gesamten Operationsteam bei Leberoperationen segmentierte DICOM-Daten zur besseren Orientierung im Sterilfeld eingespielt. Hier sollte ein genaueres Verständnis zur anatomischen Lagebeziehung von portalvenösem bzw. hepatischem Venensystem erzeugt werden. Eine von dem Projektpartner apoQlar entwickelte digitale Plattform ermöglichte es, die im VIVATOP-Projekt entwickelten Lebermodelle ähnlich der VR-Anwendung zur Operationsplanung in AR zu betrachten und damit zu interagieren. Die über dem Operationsfeld schwebenden und in den Situs hinein projizierten Modelle wurden von den Operateuren evaluiert und mit der CT-/MRT-Standarddiagnostik sowie der intraoperativen Sonographie verglichen. Die Anwendung konnte gesten- und sprachgesteuert bedient werden. Dabei hat sich in unserem perioperativen Operationsaufbau eine blaue Umgebungsbeleuchtung zur besseren Kontrastierung des AR-Modells im Bereich des Sterilfeldes als besonders nützlich erwiesen (Abb. 2). Das hat möglicherweise damit zu tun, das S‑Zapfen (blau) im Auge mit 12 % am wenigsten vertreten sind und somit das mittel- und langwellige Licht (grün, rot, gelb) der L‑ und M‑Zapfen im Hologramm noch kontrastreicher erscheinen lässt. Die VSI-Holomedicine®-Applikation ist mit einer integrierten Avatarfunktion ausgestattet, in der externe Experten zur Konsultation hinzugeschaltet werden können. Hierdurch besteht zusätzlich die Möglichkeit, die Operation live mit den augmentierten Bilddaten auf die Operationssaalbildschirme oder in Konferenzräume zu streamen.

Ein Vorteil aller immersiven Technologien ist die Möglichkeit der Zuschaltung externer Experten, die interaktiv der Operation beitreten können (Stichwort: „remote kollaborative immersive Chirurgenkonsultation“; [24]). Diese telemedizinische Anwendung wurde im Projekt zusätzlich für eine VR-Umgebung entwickelt. Mithilfe von Tiefenkameras, Sensoren und weiterer modernster Technologien werden dabei die Handbewegungen des Ärzteteams sowie die Eingriffe am operierten Organ aufgezeichnet und in VR dargestellt. Parallel können in der VR-Welt auch die Planungsdaten angezeigt werden. So ermöglichen neben den zuvor beschriebenen AR-Avatarfunktionen auch VR-Systeme eine telemedizinische kollaborative Zusammenarbeit vor komplexen Operationen.

### Training

**Abb. 3:**
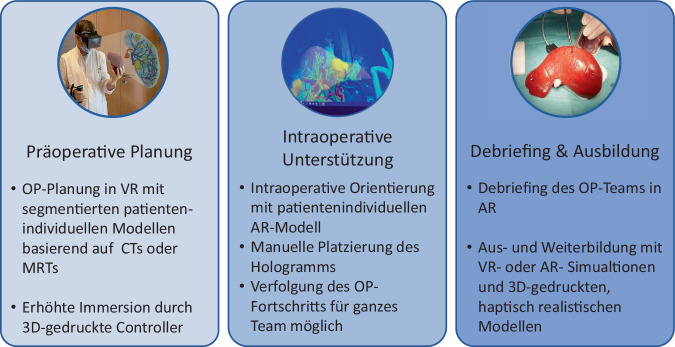
Einsatz von Virtual Realitiy (*VR*), Augmented Reality (*AR*) und 3‑D-Druck im chirurgischen Umfeld. *OP* Operation

**Abb. 4:**
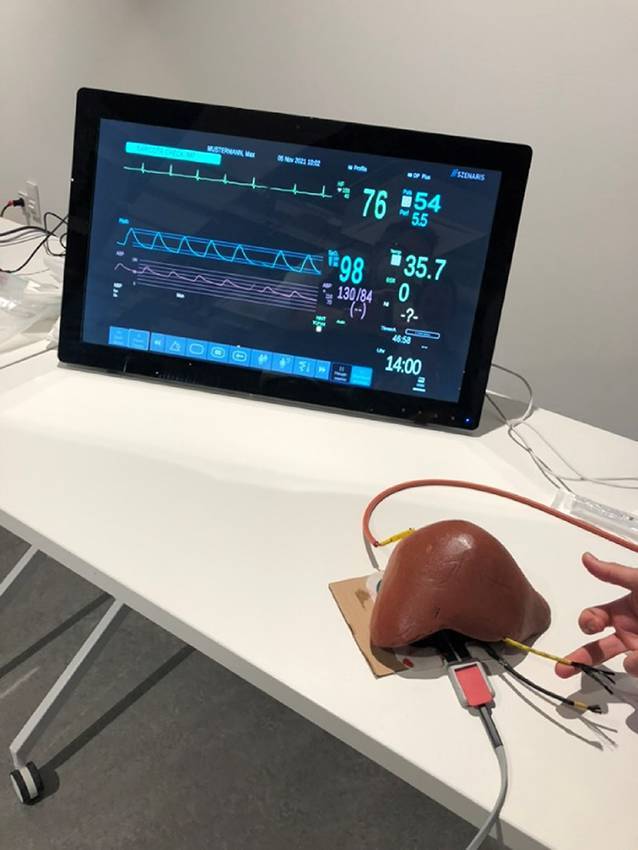
Trainingsszenario für eine Leberoperation mit haptischem Feedback

Mit der Integration von AR, VR und 3‑D-Druck soll die Aus‑, Fort- und Weiterbildung junger Ärztinnen und Ärzte effizienter gestaltet werden (Abb. 3). Konkrete Fälle sollen besser anhand der 3‑D-Bilddaten erörtert und in der virtuellen Realität präzise nachvollzogen werden [25]. Die ausgedruckten 3‑D-Modelle erlauben im Rahmen von Trainingsszenarien, bereits in einem frühen Stadium der Aus- und Weiterbildung ein „haptisches Feedback“ von Lebertumoren zu geben (Abb. 4), um diese anschließend in einer VR-Umgebung zu diskutieren [20, 21]. Zudem können einzelne Operationsschritte in virtuellen Szenarien trainiert oder reale Operationen per Telepräsenz mitverfolgt werden.

Trotz der angenommenen Vorteile in der immersiven Lernumgebung sind die Einsatzmöglichkeiten nur zum Teil evaluiert. Al Maree et al. sehen in der kollaborativen VR-Umgebung ähnlich effektive und effiziente Lernerfolge wie in der realen Lernsituationen [5]. Dennoch zeigen Ergebnisse der Arbeitsgruppe, dass weitere Forschung notwendig ist, um die effektivsten 3‑D-Interaktionstechniken und Formen der Zusammenarbeit in VR zu definieren.

### Ziel der Evaluation entwickelter Demonstratoren im klinischen Umfeld

Ziel der Studie ist die Evaluation der in VIVATOP neu- bzw. weiterentwickelten immersiven Demonstratoren in AR. Um die klinischen Ergebnisse aus dem VIVATOP-Projekt vergleichen zu können, wurden Patientinnen und Patienten, die mittels präoperativer VR-Planung und intraoperativer AR-Technologie an Lebertumoren operiert wurden, retrospektiv mit einem historischen Kollektiv verglichen. Primärer Endpunkt waren die Schnitt-Naht-Zeit (in Minuten). Sekundäre Endpunkte waren die Krankenhausverweildauer (in Tagen) der Intensivaufenthalt (in Tagen). Zudem wurden der Blutverlust, spezifische Laborparameter und die Komplikationen nach Clavien-Dindo erfasst.

## Material und Methoden

### Klinische Anwendung

Patienten, bei denen die von dem VIVATOP-Konsortium entwickelten Demonstratoren perioperativ explorativ verwendet wurden, wurden retrospektiv und monozentrisch im Matched-pair-Design mit einem historischen vergleichsweisen Kollektiv analysiert. Für die Nutzung der VR- und AR-Technologien im Rahmen von VIVATOP wurde ein Ethikvotum der Medizinischen Ethikkommission der Universität Oldenburg (Aktenzeichen 2020-153) und für die vergleichende retrospektive Studie ein weiteres Votum (2023-260) eingeholt.

Die Interventionsgruppe wurde mit der projekteigenen entwickelten VR/AR-Technologie wie vorbeschrieben geplant und intraoperativ zur Unterstützung eingesetzt. Die historische Kontrollgruppe wurde entsprechend ohne Zuhilfenahme dieser Technologien behandelt. Beide Gruppen wurden nach den bestehenden leitlinienkonformen Standard Operating Procedures (SOP) operiert. Zur Kontrollgruppe wurden alle Patienten mit Leberteilresektion bei primärem oder sekundärem Lebertumor eingeschlossen, die in einem definierten Zeitraum in der Viszeralchirurgie des Pius-Hospitals Oldenburg operiert wurden und im Krankenhausinformationssystem (KIS) erfasst sind. Zur Biaskontrolle wurden „gematchte“ Paare (z. B. Altersgruppen, Geschlecht, Grunderkrankung, Vorerkrankungen) verwendet, um Selektionsverzerrungen zu reduzieren. Es wurde ein Matching von 1:2 vorgenommen, sodass jedem Probanden oder jeder Probandin aus der AR-Interventionsgruppe zwei passende Probanden bzw. Probandinnen mit konventioneller Leberteilresektion zugeordnet werden konnte. Bei den zu „matchenden“ Lebersegmenten wurde darauf geachtet, dass bei drei betroffenen Segmenten, mindestens zwei übereinstimmen. Bei zwei oder weniger resezierten Segmenten, stimmte mindestens ein Lebersegment überein. Zum Vergleich der Grunderkrankung wurden die ICD(International Statistical Classification of Diseases and Related Health Problems)-10-Codes herangezogen.

Einschlusskriterien für die AR-Interventionsgruppe waren eine Indikation zur Operation bei primären oder sekundären Lebertumoren/-metastasen, die Indikation zur offenen Leberteilresektion und Einwilligung zur Studienteilnahme an der VIVATOP-Studie. Für das historische Kollektiv wurden Patientinnen und Patienten mit übereinstimmenden ICD-10 und OPS-Codes eingeschlossen.

Ausgeschlossen wurden Patientinnen und Patienten, die für die Kontrollgruppe nicht vergleichbare Lebersegmente oder nicht matchende biometrische Voraussetzungen erfüllten.

### Präoperative Planung und intraoperative Unterstützung in der Interventionsgruppe

**Abb. 5:**
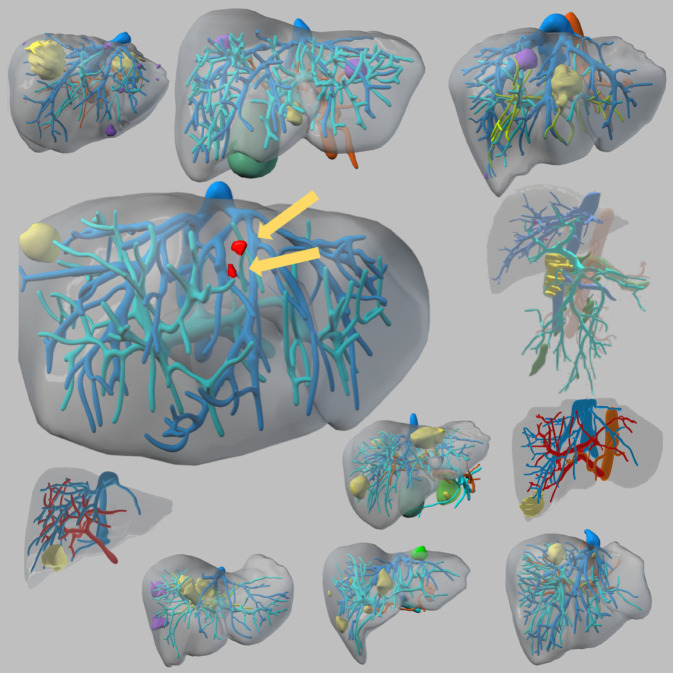
Darstellung der segmentierten Lebern aus dem VIVATOP-Projekt. Das große Modell zeigt zwei mittels künstlicher Intelligenz (*KI*) identifizierte Raumforderungen (*Pfeile*) unklarer Dignität (*rot*)

Präoperativ wurde der oben beschriebene, im Rahmen von VIVATOP entwickelte VR-Demonstrator zur Operationsplanung genutzt. Abb. 5 zeigt die 11 segmentierten 3‑D-Lebermodelle der betroffenen Patientinnen und Patienten. Alle an der Operation beteiligten Chirurginnen und Chirurgen haben sich diese segmentierten Modelle in der VR-Umgebung zur Operationsvorbereitung angeschaut und zusätzlich zu CT/MRT-Standarddiagnostik virtuelle Resektionsplanungen durchgeführt. Intraoperativ wurde im Rahmen der konventionellen Leberteilresektion vor der Resektionsphase das segmentierte 3‑D-Bild sowohl über den Operationssitus eingeblendet als auch in den Situs hineinprojiziert. Die Resektion der Tumoren und KI(künstliche Intelligenz)-detektierten Läsionen erfolgte dann standardisiert gemäß den leitlinienkonformen SOPs und nur in einem definierten Zeitfenster unter Zuhilfenahme der Hololens II zur intraoperativen Orientierung. Während der gesamten Operation war eine technisch speziell eingewiesene Studienassistenz anwesend.

### Statistik

**Abb. 6:**
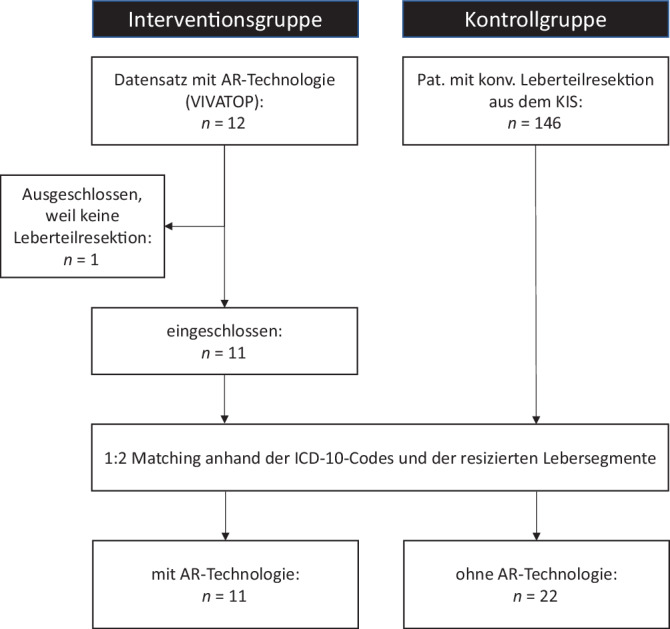
Flowchart zum Vergleich der Augmented-Reality(*AR*)-Interventionsgruppe vs. Kontrollgruppe. *ICD-10 *International Statistical Classification of Diseases and Related Health Problems 10, *KIS* Krankenhausinformationssystem

Die Datenauswertung erfolgte EDV-gestützt mittel SPSS (IBM Deutschland GmbH, Ehningen). Die Daten wurden als Matched-pair-Analyse ausgewertet. Von den insgesamt 12 im Projektzeitraum mit AR-/VR-Unterstützung operierten Patientinnen und Patienten konnten 11 in die Interventionsgruppe eingeschlossen werden (Abb. 6). Aus den 146 Patientinnen und Patienten, die aus dem KIS vergleichsweise identifiziert wurden, wurden 22 entsprechend in die Kontrollgruppe anhand der Grunderkrankung (ICD-10-Code) und des resezierten Lebersegments im Verhältnis 1:2 „gematcht“.

Zur deskriptiven Auswertung der Patientencharakteristika Alter (Jahre), Geschlecht, Größe (cm), Gewicht (kg) und Body-Mass-Index (BMI; kg/m^2^) wurden der Mittelwert, das Minimum, das Maximum und die Standardabweichung berechnet. Für die Analyse auf signifikante Unterschiede zwischen den beiden Gruppen bezüglich der primären und sekundären Endpunkte wurde der Mann-Whitney-U-Test genutzt. Ein *p*-Wert von 0,05 wurde als Signifikanzniveau angenommen. Die Parameter Blutverlust, Laborwerte und Komplikationen wurden rein deskriptiv ausgewertet.

## Ergebnisse

### Patientencharakteristika

Die beiden Patientengruppen waren basierend auf den von uns ausgewählten Variablen zur Beschreibung der Patientencharakteristika sehr vergleichbar (Tab. 1). In der AR-Interventionsgruppe litt jede Patientin bzw. jeder Patient an mindestens einer Vorerkrankung. Am häufigsten waren kardiovaskuläre Erkrankungen, wie z. B. arterielle Hypertonie mit 14,5 % (*n* = 8). Sonstige Erkrankungen gaben 10,9 % (*n* = 6) der Probanden an, metabolische Vorerkrankungen wie Diabetes mellitus Typ 2 lagen in 7,3 % (*n* = 4) vor. Es lagen insgesamt 24 Voroperationen bei den Probanden vor, davon waren mit 33,3 % (*n* = 8) der Nennungen der Kategorie sonstige allgemeinchirurgische Eingriffe zuzuordnen. Am zweithäufigsten (16,7 %) wurden Darmteilresektionen durchgeführt. In der Kontrollgruppe sind kardiovaskuläre Vorerkrankungen (19,3 %) und gastroenterologische Erkrankungen (14,0 %) am häufigsten. In der Kontrollgruppe wurden 38 Voroperationen erfasst. Auch hier zählten die sonstigen allgemeinchirurgischen Operationen mit 10 Nennungen (26,3 %) und Darmteilresektionen mit 8 Nennungen (21,1 %) zu den häufigsten.

**Tab. 1:** Deskriptive Beschreibung des Patientenkollektivs

		Interventionsgruppe	Kontrollgruppe
	*n*	11	22
Alter (Jahre)	Mittelwert	71,4	69,1
	Standardabweichung	7,5	8,3
	Minimum	58	54
	Maximum	80	83
Größe (cm)^a^	Mittelwert	170,2	172,5
	Standardabweichung	8,8	10,2
	Minimum	156	151
	Maximum	178	190
Gewicht (kg)^b^	Mittelwert	78	83,1
	Standardabweichung	10,12	15,99
	Minimum	67,7	50
	Maximum	96,9	111
BMI (kg/m^2^)^c^	Mittelwert	27	27,68
	Standardabweichung	3,24	3,92
	Minimum	21,62	20,43
	Maximum	30,93	35

*n* = Größe der Stichprobe

^a^Gültige Werte: Interventionsgruppe (*n* = 11), Kontrollgruppe (*n* = 20), fehlende Werte: Interventionsgruppe (0), Kontrollgruppe (2)

^b^Gültige Werte: Interventionsgruppe (*n* = 11), Kontrollgruppe (= 21), fehlende Werte: Interventionsgruppe (0), Kontrollgruppe (1)

^c^Gültige Werte: Interventionsgruppe (*n* = 11), Kontrollgruppe (*n* = 20), fehlende Werte: Interventionsgruppe (0), Kontrollgruppe (2)

### Primäre und sekundäre Endpunkte

Schnitt-Naht-Zeit (*p* = 0,665), die Krankenhausverweildauer (*p* = 0,317) und die Dauer des Intensivaufenthalts (*p* = 0,063) unterscheiden sich nicht zwischen Interventions- und Kontrollgruppe (Tab. 2).

**Tab. 2:** Perioperative Variablen. (Angegeben sind Mittelwerte und Range)

	AR-Interventionsgruppe	Kontrollgruppe
Schnitt-Naht-Zeit (Minuten)	140 (114,9, 164,9)	138 (125,2,150,9)
Krankenhausverweildauer (Tage)	16 (6,1, 25,7)	16 (11,4, 19,8)
Intensivstationsverweildauer (Tage)	4 (1,2, 6,7)	5 (2.6, 6,9)

### Intraoperativer Blutverlust und Laborparameter 2. bis 4. postoperativer Tag

Der intraoperative Blutverlust war bei der Kontrollgruppe im Durchschnitt mit 650 ml (SD: 269 ml) niedriger als in der Interventionsgruppe (MW: 456 ml; SD: 270 ml). Die leberspezifischen Laborparameter zwischen dem 2. bis 4. postoperativen Tag Aspartat-Aminotransferase (AST), Alanin-Aminotransferase (ALT) und γ‑Glutamyltransferase (GGT) waren in beiden Gruppen erhöht (Tab. 3). In der AR-Interventionsgruppe zeigte sich eine tendenziell geringere Erhöhung der leberspezifischen Laborparameter. Auffällig ist das beinahe doppelt höhere C‑reaktive Protein (CRP) in der Kontrollgruppe im Vergleich zur AR-Interventionsgruppe.

**Tab. 3:** Laborparameter

		Interventionsgruppe	Kontrollgruppe	Normwerte^a^
		*n* = 11	*n* = 22	
Bilirubin (mmol/l)	Mittelwert	0,0109	0,0193	< 0,0188 mmol/l
	Median (Min., Max.)	0,0086 (0,0034, 0,0325)	0,0077 (0,0034, 0,1539)	–
AST (IU/l)	Mittelwert	114,73	154,68	< 50 IU/l
	Median (Min., Max.)	91,0 (32, 271)	125,5 (22, 402)	–
ALT (IU/l)	Mittelwert	179	308,36	< 50 IU/l
	Median (Min., Max.)	128,0 (75, 303)	246,00 (37, 812)	–
GGT (IU/l)	Mittelwert	81,55	113,36	< 71 IU/l
	Median (Min., Max.)	68,0 (16, 219)	101,00 (18, 270)	–
Alkalische Phosphatase (IU/l)	Mittelwert	96,09	102,23	40−129 IU/l
	Median (Min., Max.)	98,00 (46, 160)	89,00 (44, 240)	–
LDH (IU/l)	Mittelwert	222,64	236,95	< 225 IU/l
	Median (Min., Max.)	217,00 (150, 334)	245,00 (150, 312)	–
Cholesterinesterase (IU/l)2	Mittelwert	3959	3421,29	530–1290 IU/l
	Median (Min., Max.)	3959,00 (2985, 4933)	3330,00 (1922, 4884)	–
Alpha-Amylase (IU/l)	Mittelwert	29,09	35,36	28–100 IU/l
	Median (Min., Max.)	27,0 (12, 44)	28,50 (9, 75)	–
Lipase (IU/l)	Mittelwert	25,45	20,77	< 60 IU/l
	Median (Min., Max.)	20,0 (9, 79)	19,00 (8, 44)	–
C‑reaktives Protein (mg/dl)^b^	Mittelwert	11,97	21,75	< 5,0 mg/dl
	Median (Min., Max.)	9,1 (4,8, 31,0)	13,25 (4,7, 138,0)	–

*ALT* Alanin-Aminotransferase, *AST* Aspartat-Aminotransferase, *CRP* C-reaktives Protein, *GGT* γ-Glutamyltransferase, *LDH* Laktat-Dehydrogenase

^a^https://elibrary.kohlhammer.de/book/10.17433/978-3-17-026444-1/download?

^b^Gültige Werte Interventionsgruppe (*n* = 2), gültige Werte Kontrollgruppe (*n* = 7)

### Komplikationen nach Clavien-Dindo

**Abb. 7:**
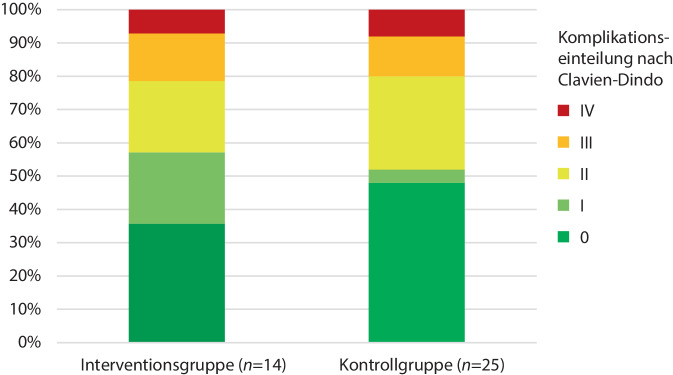
Darstellung der Komplikationsraten für die Interventions- und die Kontrollgruppe in Abhängigkeit von der Komplikationsschwere nach Clavien-Dindo

Bei 45,5 % (5/11) der mit AR-Technologie operierten Probanden kam es zu keinen Komplikationen, in der Kontrollgruppe verliefen 54,5 % (12/22) der Eingriffe komplikationsfrei. Da es bei einigen Probanden zu mehrfachen Komplikationen kam, traten in der AR-Interventionsgruppe insgesamt *n* = 14 und in der Kontrollgruppe insgesamt *n* = 25 Komplikationen auf. Nach Clavien-Dindo eingeteilt fand sich kein Unterschied zwischen den beiden Gruppen, speziell auch bei höheren Komplikationsgraden (Abb. 7). Komplikationen nach Clavien-Dindo Grad II waren fast ausschließlich der Einsatz systemischer Antibiotikatherapie im Rahmen beginnender bronchopulmonaler Infektionen. Grad-V-Komplikationen traten in keiner der beiden Gruppen auf.

## Diskussion

Dreidimensionale Visualisierungstechnologien wurden bereits vor über 20 Jahren als intuitive technologische Weiterentwicklungen beschrieben, die es Chirurgen ermöglichen, virtuelle Operationsplanungen durchzuführen, um das Lebervolumen zu berechnen und so maßgeblich das klinische operative Vorgehen vorzubereiten [26]. Die Anwendung der dreidimensionalen Visualisierung bei Leberresektionen befindet sich seitdem in einer stetigen Entwicklungsphase und bisher fehlen klinisch-evidenzbasierte Beweise dafür, dass durch eine dreidimensionale Visualisierung die Patientensicherheit und das klinische Outcome nach Leberresektionen verbessert werden.

Eine kleine Fallserie von Mero et al. zeigt verlängerte Operationszeiten bei Leberresektionen bei Einsatz von AR-Technologie. Diese lagen mit durchschnittlich mit 189,5 min deutlich über dem Mittelwert der ermittelten durchschnittlichen Operationsdauer [18]. Als vorteilhaft sahen die Autoren jedoch ein verbessertes Anatomieverständnis beim Einsatz der AR-Technologie. In unserer vergleichenden Fallserie konnten wir dagegen keinen signifikanten Unterschied bez. der Operationszeit, der Krankenhausverweildauer und dem Intensivaufenthalt zwischen der AR-Interventionsgruppe und der Kontrollgruppe nach Leberteilresektionen feststellen. In Bezug auf die Verwendung von Blutprodukten und den Blutverlust zeigte sich ein diskreter Vorteil in der AR-Interventionsgruppe. In anderen Studien mit vergleichbaren Prozeduren lag der durchschnittliche Blutverlust bei 650 ml und damit im Bereich unserer Kontrollgruppe ca. 200 ml höher als in unserer AR-Interventionsgruppe [27]. Belastbare klinische Studien mit intra- oder postoperativen Outcomes wie Operationsdauer oder Komplikationsraten gibt es bisher kaum.

Dass eine intraoperative dreidimensionale Darstellung des Situs mit speziellen 3‑D-Kameras in der Leberchirurgie potenzielle Vorteile bietet, belegen Zhang et al. in einer neueren Metaanalyse [28]. In der 3‑D-Gruppe fand sich sogar eine kürzere Operationszeit (mittlere Differenz [MD] = 34,39; 95 %-Konfidenzintervall[KI] = 59,50, 9,28), ein geringerer intraoperativer Blutverlust (MD = 106,55; 95 %-KI = 183,76, 29,34) und ein geringeres Bluttransfusionsvolumen (MD = 88,25; 95 %-KI = 141,26, 35,24). Die 3‑D-Gruppe wies zudem eine geringere Differenz zwischen dem vorher prognostizierten und dem klinisch tatsächlich resezierten Volumen auf (MD = 103,25; 95 %-KI = 173,24, 33,26). Zudem fand sich eine geringere Rate an postoperativen Komplikationen (Odds Ratio [OR] = 0,57; 95 %-KI: 0,35, 0,91).

Die Morbidität bei Lebereingriffen wird in der Literatur um die 45 % angegeben. Die häufigsten Komplikationen sind intra- oder postoperative Blutungen, Leberversagen, Leberabszesse, Biliome und Gallenleckagen [29]. Laut Studienübersicht entwickeln 11 % eine schwerwiegendere Komplikation, wozu respiratorische Insuffizienz (4,9 %), Sepsis (6,1 %), Nierenversagen, Myokardinfarkt, Herzstillstand und Lungenembolien gezählt werden [30]. In der VIVATOP-Evaluation kamen die Grad IV Komplikationen nur in 7,1 % der Fälle vor, verglichen zur Kontrollgruppe (8,0 %) und liegen in beiden Gruppen unterhalb der 11 % der Grad-IV-Komplikationsrate aus anderen publizierten Studien [30].

In einer aktuellen Literaturübersicht wurden *n* = 102 Studien und Reviews zusammengefasst, die sich mit dem Einsatz von AR-Technologie im Rahmen von Leberchirurgie beschäftigen [7]. In 28 Publikationen wird über 183 Patienten berichtet, die mithilfe von AR-Technologie durch Laparotomie (*n* = 31) oder Laparoskopie (*n* = 152) operiert wurden. Dabei war die anatomische Präzision das wichtigste Bewertungskriterium in 19 von 28 Artikeln. Die Autoren kommen zu dem Schluss, dass fast 20 Jahre nach der ersten Verwendung von AR in der Neurochirurgie die AR-Technologie in der Leberchirurgie im Wesentlichen auf die Forschung beschränkt ist. Dieses steht allerdings ganz im Gegensatz zur orthopädischen Chirurgie und Neurochirurgie, bei denen AR-Technologien bereits in der klinischen Routine eingesetzt werden.

AR-Technologien bewegen sich weg vom experimentellen Setting hin zu klinischen Studien

Ob in der Viszeralchirurgie wie in unseren Evaluationen die tendenziell günstigeren postoperativen Leberfunktions- und Entzündungsparameter in Zusammenhang mit der eingesetzten AR-Technologie stehen, darf bezweifelt werden, da einerseits nur eine kleine Stichprobe eingeschlossen werden konnte und der Vergleich mit einem historischem Patientenkollektiv erfolgte. Dennoch lässt sich feststellen, dass sich digitale Assistenzsysteme wie die AR-Technologien scheinbar an einem Wendepunkt befinden. Eine zunehmende Anzahl von Technologien bewegt sich weg vom „proof of concept“ in experimentellen Settings hin zu klinischen Studien wie auch im vorliegendem Bericht [1]. Dabei ist ein Trend erkennbar, der sich voraussichtlich beschleunigen wird, da der technologische Fortschritt in der z. T. vollautomatischen Segmentierungssoftware die bestehenden Workflowprobleme gelöst hat, Kosten senkt und damit der breite Zugang dieser Technologien in der klinischen Routine zunehmend vorstellbarer wird [2].

Während in der medizinisch-anatomischen Lehre bereits ein deutlicher Mehrwert von VR/AR Unterstützungssystemen nachgewiesen ist [31–33], muss in der klinischen Anwendung ein überzeugender Mehrwert weiterhin nachgewiesen werden. In dem Verbundprojekt VIVATOP mit Partnern aus der Industrie wie CIRP und Szenaris, dem Fraunhofer-Institut MEVIS und dem Technologiezentrum für Informatik und Informationstechnik (TZI) der Universität Bremen wurden digitale Assistenzsysteme durch neue Softwarelösungen für VR/AR und 3‑D-Druck neuentwickelt und bestehende Technologien weiterentwickelt. Die Evaluation der entsprechenden Demonstratoren umfasste die präoperative Planung, intraoperative Unterstützung und postoperative Ausbildung an patientenrealistischen Fällen. Die im Projekt angewendeten halbautomatischen KI-basierten Segmentierungen zeigen bespielhaft das Potenzial dieser Softwareentwicklungen anhand des in Abb. 5 dargestellten Modells. Die im CT/MRI nicht sichtbaren Läsionen konnten intraoperativ in Kombination mit der Hololens II und der Bilddatenprojektion in den Operationssitus leicht identifiziert werden. In dem Fallbeispiel handelte es sich um histologisch subkapsuläre Fibrosen, die allerdings auch eine sehr frühe fein granuläre Metastasierung hätten sein können.

Die Operationszeit wird durch den Technologieeinsatz nicht verlängert

Auch wenn sich im Verlauf der operativ-technischen Weiterentwicklungen die Morbidität < 45 % und Mortalität < 5 % in der Leberchirurgie senken ließ [29], so kann für digitale Unterstützungstechnologien zumindest festgehalten werden, dass eine subjektiv viel genauere Vorstellung der anatomischen Beziehung der Tumoren zur Gefäßsituation und deren Normvarianten für das gesamte Team erreicht wird. Zudem scheint in Bezug auf objektivierbare Parameter mindestens Gleichwertigkeit zu den konventionellen Verfahren zu bestehen, ohne die Operationszeit durch den Technologieeinsatz zu verlängern. In der Aus- und Weiterbildung an den patientenrealistischen Fällen konnte unter Verwendung der oben beschrieben VIVATOP-Segmentierungen gezeigt werden, dass die AR-Technologie den konventionellen CT/MRT-Bildgebungen in Bezug auf die Korrektheit und die Geschwindigkeit der Diagnosestellung weit überlegen sind [33].

### Limitationen

Eine Limitation der hier beschriebenen Demonstratorenerprobung ist die projektbedingt geringe Stichprobe sowie der Vergleich mit einem historischen Kollektiv. Eine weitere Limitation ist, das geplante bizentrische Evaluationen pandemiebedingt nicht bzw. nur eingeschränkt erfolgen konnten. Zukünftig sollten weitere multizentrische und interprofessionelle klinische Studien folgen, um vollautomatisierte Visualisierungssoftware zu entwickeln und den klinischen Nutzen im Sinne eines verbesserten klinischen Outcomes und damit einer höheren Patientensicherheit nachzuweisen.

## Fazit für die Praxis

- Digitale Assitenzsysteme wie Augmented (AR) und Virtual Reality (VR) scheinen in der Viszeralchirurgie an einem Wendepunkt zu stehen.
- Auf künstliche Intelligenz basierte automatische Softwarelösungen bieten schon heute eine schnelle Verfügbarkeit der dreidimensionalen Bildsegmentierung, die in Aus- und Weiterbildung zu einem signifikant besseren anatomischen Verständnis führen.
- Bei gründlicher Operationsplanung in VR verlängert sich die Operationszeit trotz intraoperativer AR-Technologieanwendung nicht.
- Die klinischen Ergebnisse zeigen trotz kleiner Stichproben sowohl in der Literaturevidenz als auch in der eigenen Fallserie subjektive (Anatomieverständnis) und objektive Vorteile im klinischen Outcome.
- In der Viszeralchirurgie und der Modellprojektion in das operative Sterilfeld müssen für eine klinische Routineanwendung die Registrierung der segmentierten Bilder und das Problem der intraoperativen Organdeformation gelöst werden.
